# Impact of Job Insecurity on Hotel Workers’ Workaholism and Work–Family Conflict in Korea

**DOI:** 10.3390/ijerph17217783

**Published:** 2020-10-24

**Authors:** JaeWon Shin, HyoungChul Shin

**Affiliations:** 1 Department of Tourism Management, Kyonggi University, Seoul 033746, Korea; sjw740@hanmail.net; 2 Department of Foodservice and Culinary Management, Kyonggi University, Seoul 033746, Korea

**Keywords:** job insecurity, workaholism, work–family conflict, hotel industry, human resource management

## Abstract

This study explored the relationship between job insecurity of employees and workaholism or work–family conflict in the hotel industry in Korea. To do this, four hypotheses were proposed. First, that job insecurity will have positive effects on workaholism. Second, that workaholism will have positive effects on work–family conflict. Third, that job insecurity will have positive effects on work–family conflict. Fourth, that through the mediation of workaholism, job insecurity will have positive effects on work–family conflict. Further, eligible respondents (*n* = 331; 217 male and 112 female) were recruited from four-star hotels or above located in Seoul, Incheon, and Gyeonggi Province and then evaluated for a self-administered questionnaire survey. Results showed that job insecurity had significant positive effects on workaholism, and workaholism had significant positive effects on work–family conflict and mediated the interaction between job insecurity and work–family conflict. Thus, it can be concluded that hotels should improve working conditions and propose solutions, such as the moderation of workload, for preventing their workers from workaholism. In particular, hotel business managers should minimize worker’s job-insecurity-induced compulsive drive to work by devising strategies for minimizing their worker’s workloads. They should also enable workers to perform their jobs autonomously.

## 1. Introduction

Job insecurity has continued to cause social problems in Korean society since the financial crisis of 1997. Firms are continuously required to manage their organizations effectively with the minimum amount of human resources possible to survive in a rapidly evolving business environment. Moreover, unpredictable market economy conditions and intensified competition among businesses have led to the transformation of corporate structures, for example, downsizing and mergers and acquisitions; such transformations within the job market are exacerbating the problem of job insecurity [1].

These changes in the business environment have a profound impact on hotel businesses as well. Particularly, hotel businesses have come to formulate diverse business strategies and methods to ensure their survival by proposing more proactive solutions that reflect changes in the job market. Mainly, hotels have laid off workers and replaced them with low-wage, temporary workers. Additionally, with the exception of key departments, they have outsourced jobs in various fields. As a result, the job security of hotel workers has diminished, and it has continued to have a serious psychological effect even on those workers who have managed to keep their jobs [2]. Job insecurity, which stems from constant changes in the working environment, is affecting workers in various arenas; it has been exerting a long-term influence on their work attitudes and behaviors [3]. Moreover, as workers immerse themselves in their work and work for long hours to give the impression of having important roles, they may become workaholics [4,5].

In addition to job insecurity, workers experience longer working hours and increased performance pressure in a competitive society [6]. Complex interrelated industrial advancements have also blurred the boundaries among different types of work. Such developments led to the emergence of the concept of “workaholism” that refers to the phenomenon of being so preoccupied by one’s work as to lose self-control and neglect other areas of one’s life. Organizations may support workaholism because work has long been touted as a virtue. Nonetheless, like any other addiction, the symptomatic anxiety of workaholism experienced when not working can have a negative impact not only on a workaholic’s physical and mental health but also on his or her family [7,8]. In contrast to Western societies, Korean society has an extremely low level of awareness regarding the problems of workaholism, which is accompanied by a structure that is susceptible to workaholism. Workaholism is portrayed in a good light because outperforming expectations in a given role are equated with being devoted to one’s family and company [9]. In such a culture where workers must work long hours to exceed company goals, Korean workers are likely to become workaholics. However, working for long hours does not necessarily imply workaholism. According to the data released in 2018, Koreans worked for an average of 1,993 h, which was second only to the number of hours worked by Mexicans [10].

Work and family are two important areas in an individual’s life, and in addition to job insecurity, their correlation with workaholism can affect family relationships. Consequently, it may not be enough to investigate the correlation between job insecurity and workaholism separately from the impact of work–family conflict. Moreover, there prevails a correlation between workaholism and work–family conflict, and several previous studies have revealed that workaholism has a negative impact on family relationships [11,12,13].

To date, most of the studies conducted on job insecurity or employment instability have focused on investigating the correlations among factors such as organizational commitment, work-related stress, job performance, and turnover intention. Little research has been conducted to investigate how perceived job insecurity has affected workaholism and work–family conflict.

Thus, this study empirically verifies not only the impact of hotel workers’ perception of job insecurity on workaholism (driven by the need for approval by the organization) and work–family conflict but also the mediating effects of workaholism on job insecurity and work–family conflict. We also verify the effects of job insecurity on work–family conflict by mediating workaholism. It thereby hopes to play a part in reducing the negative effects of job insecurity and workaholism in the hotel industry. Additionally, it intends to offer implications for how hotels can manage their workers effectively, thereby maximizing the efficiency of human resource management.

## 2. Theoretical Background

### 2.1. Job Insecurity

Perceived by workers as a chronic potential threat in the workplace [14], job insecurity is a topic that has been studied by several different scholars [3,15,16,17]. Job insecurity indicates a sense of powerlessness that arises from the failure of safeguarding a desired job in the face of unstable employment conditions [18]. It can be characterized as a subjective phenomenon that is based on the cognitive assessments of a risky employment situation. Perceived job insecurity is categorized, first, in the sense of threat of job loss, and second, in the sense of a feeling of powerlessness from being unable to deal with that threat. In the first sense, the threat of the loss of one’s primary function or the job itself serves as the source of job insecurity, and in the second sense, the sense of powerlessness is considered an important factor because it intensifies the sense of threat experienced [15].

Such diverse definitions and operationalizations are examined with respect to job insecurity in the four following ways:

1. job insecurity in the social sense that includes a general awareness of a high unemployment rate;
2. job insecurity in the organizational sense that derives from a company’s reference to unstable and risky working conditions;
3. an intense job insecurity that encompasses subjective experiences that threatens one’s job;
4. job insecurity as the anticipation of job loss in a situation where a company has already begun laying off employees [19].

It can thus be concluded that job insecurity is an experience caused by individual or external influences [20]. Comparative studies of objective and subjective job insecurity have revealed that compared to temporary workers, permanent workers with low job satisfaction and low work commitment experience a higher level of job insecurity [21]. As such, the task of defining job insecurity requires an examination of the nature of the circumstances itself and the subjective factors at play.

Organizational transformations, such as restructuring and mergers, have not only caused many workers to lose their jobs but also significantly reshaped the jobs performed by those who keep their jobs [22,23]. Those who have experienced restructuring in the past will also expect additional organizational changes in the distant future and perceive their own employment status as being at risk. Moreover, being placed in a new role after restructuring causes role ambiguity, which heightens workers’ awareness of this other sense of job insecurity.

Various studies have been conducted on the factors that have a direct impact on job insecurity, and the most common factors are age, gender, personal disposition, socioeconomic situation, form of employment, and social support [15,19,21,24,25,26]. Furthermore, the effects of workaholism include low job performance, low work commitment, low job satisfaction, reduced trust, rate of absenteeism, turnover intention, work effort, and resistance to change [5,18,27,28,29].

### 2.2. Workaholism

Previously, the term “workaholism” only applied to people who simply worked for long hours, but it has come to encompass people who are motivated by the act of working for long hours itself [30]. While long working hours is typically used to gage workaholism, there have recently been signs in the workplace that this perception is evolving. Even if there is a correlation between workaholism and working hours, working hours cannot be an ideal factor for measuring workaholism.

Coined in a study drawing similarities between workaholism and alcoholism, workaholism has been defined as becoming obsessed with work and losing one’s work–life balance [31]. Characterized as becoming irrationally and excessively engrossed in work [32], workaholism was deemed a mental disorder accompanied by severe compulsions [33].

While workaholism is viewed as an obsession with one’s work, some researchers have asserted that it has a positive impact on workers and companies in the short run, even as others have contended that in the long run, it means reduced productivity for the companies and psychological deterioration for the workers [34,35,36,37,38].

Workaholism is a complex idea constituting several different components, and thus, workaholism can be measured in numerous ways [39]. Moreover, some studies treat workaholism as an addiction [40], a behavioral pattern [41], a work attitude [42], or a symptom [43]. However, the methods of measuring workaholism that consider excessive work commitment and low enjoyment and, in so doing, overcome the shortcomings of the existing studies that possess one-dimensional approaches to classify the different types of workaholics [42] are the most popular [44]. The three following concepts were devised for the classification of workaholism:

- The first concept was work commitment, dealing with the notion that workers must be productive during their working hours.
- The second concept was being driven to work that relates to a sense of duty.
- The third concept was work enjoyment, which is enjoyment derived from performing a task.

Since then, numerous researchers [36,45,46,47,48] have used the abovementioned concepts to measure workaholism.

This study employs the most popular metrics with proven validity and reliability to measure workaholism [42]. However, it does not explore the concept of work enjoyment, as it has concluded that it is difficult for workers who are experiencing job insecurity to take pleasure in their work, i.e., experience work enjoyment.

### 2.3. Work–Family Conflict

Previously, a strong tendency prevailed in relation to distinguishing work life from family life [49]. At present, however, these two are recognized as sharing the same interface because they act upon each other [50]. Work–family role conflict is defined as the conflict that arises when the different roles within an individual’s work life and family life clash [51]. In other words, such conflict between the two interrelated roles may be defined as a form of conflict of roles that occurs when pressures on the roles within the work and family lives contradict each other in some way [52]. It is also considered as a form of conflict that occurs when the pressures on the roles within each of the areas of work life and family are incompatible [53].

The effects of work–family conflict have been studied from structural and psychological viewpoints. Structural attributes include working hours and overtime, and psychological attributes include job characteristics and work overload. The physical and psychological fatigue resulting from said attributes were found to have a negative impact, thus making it difficult to fulfill the expected family roles [54,55].

As explained above, most of the studies on work–family conflicts treat work–life conflict as a form of conflict between the two roles within the work life and family life [49,52,56,57,58,59,60]. This study also posits that the variables for work–life conflict are different from each other, and it adopts the widely-used metrics identified by Greenhaus and Beutell [52] to measure them.

### 2.4. Correlation among the Variables

From the standpoint of the organization, perceived job insecurity is observed to elicit different behavioral reactions from workers. The most common observation is that workers are stressed by the idea of staying with their organizations, leading to high turnover rates [15,17,28,61,62,63]. Other studies have highlighted that workers who perceive a risk of a layoff increase their own workload in an effort to convince their organizations that they are valuable assets [4,64]. On the basis of the findings of such studies, this study developed its first hypothesis:

**Hypothesis** **1.** *Job insecurity will have positive effects on workaholism.*

As the level of workaholism increases, there exists a proportionate increase in the perception of family dysfunction, impairing family engagement and communication [65,66]. Work–family conflict reduced life satisfaction of workaholics [45], and serious workaholics experienced a work–life imbalance and faced troubles while finding some time for their families [67,68]. On the basis of the findings of such studies, this study developed the second hypothesis:

**Hypothesis** **2.** *Workaholism will have positive effects on work–family conflict.*

Job insecurity has a strong psychological impact on workers, and eventually, it will have serious consequences throughout their whole lives [15,27]. A study of double-income households concluded that job security is crucial to workers and that corporate restructuring has a severe negative impact on the ability to lead an ordinary family life [55,69]. A greater perception of job security by workers generally reduced work–life conflict and turnover intention [70]. On the basis of the findings of such studies, this study developed the third hypothesis:

**Hypothesis** **3.** *Job insecurity will have positive effects on work–family conflict.*

Finally, this study constructed the fourth hypothesis to verify the mediating effects of workaholism on job insecurity and work–family conflict.

**Hypothesis** **4.** *Through the mediation of workaholism, job insecurity will have positive effects on work–family conflict.*

**Figure 1:**
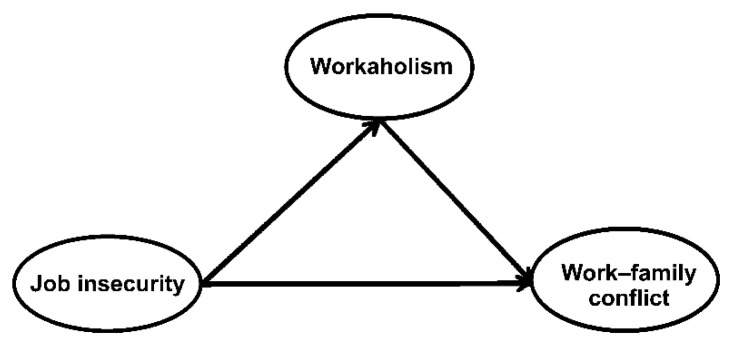
Study model.

As shown in Figure 1, the current study examined whether job insecurity on hotel workers’ and work–family conflict.

## 3. Materials and Methods

### 3.1. Data Collection and Method

Using the convenience sampling method between October 1st and 31st in 2019, this study conducted a field survey of a sample of hotel workers who worked in four-star hotels or above in Seoul, Incheon, and Gyeonggi Provinces. We explained to the participants the details of our study and asked for permission to collect data. The participation would be voluntary and anonymous, guaranteeing confidentiality.

To perform an empirical analysis, 400 questionnaires were distributed, 350 copies of which were reclaimed. Of the reclaimed copies, 19 copies whose responses were judged to be unreliable were excluded, resulting in the use of 331 copies for empirical analysis. To test the theoretical model designed, the study used SPSS 18.0 (IBM, Armonk, NY, USA) to perform an exploratory factor analysis. It then performed a reliability analysis for each factor and conducted a confirmatory factor analysis using AMOS 18.0 (IBM, Armonk, NY, USA) to verify the conceptual independence of this study. Further, it performed a correlation analysis to verify the direction and discriminant validity of the hypotheses, and then, structural equation modeling was employed to test the hypotheses.

### 3.2. Measurement

The questionnaire was designed by revising and supplementing the insights of previous studies on job insecurity, workaholism, and work–family conflict. To measure job insecurity, various studies were evaluated [27,64], and a method of measurement was designed through revision and supplementation. The responses to a total of five questions, such as “I am not sure when I might be asked to leave the company” and “I am anxious about the possibility of another layoff in the future”, were evaluated on a 5-point Likert scale. The survey questions used in the empirical study conducted by Spence and Robbins [42] were revised to measure workaholism in this study. Of the sub-factors that form the concept of workaholism, only work commitment and the compulsive drive to work were evaluated; work enjoyment was excluded. The responses to a total of 12 questions, including “I get bored when I am not working” and “I have an obsessive drive to work”, were evaluated on a 5-point Likert scale. As for the measurement of work–family conflict, the methods of measurements adopted in previous studies [57,58,71] were revised and supplemented. The responses to a total of six questions, such as “When I get off work, I’m too tired to do what I want at home” and “I am too busy with work to spend time with family and friends”, were evaluated on a 5-point Likert scale.

## 4. Results

### 4.1. Demographics of the Participants

The demographics of the participants are illustrated in the following manner. Males and females constituted 65.6% and 34.4% of the sample, respectively. In terms of age, the majority of the participants were in their 20s, accounting for 43.2% of the sample size. In terms of education, 58.3% held associate degrees. As for experience, 45.3% had less than 5 years of experience, and 26.6% worked in the food and beverage departments. Their demographic factors of the participants are summarized in Table 1.

### 4.2. Analysis of the Reliability and Validity

#### 4.2.1. Confirmatory Factor Analysis

The results of the confirmatory factor analysis performed to prove the validity of this study’s structure are presented in Table 2. Workaholism, which consists of secondary factors in this study, was parameterized as a primary factor after finding the average of the sub-variables evaluated. As a result, the following results were obtained, and the model was deemed to be acceptable: χ^2^ = 154.187 (df = 62, *p* = 0.000), CMIN/DF = 2.487, RMR = 0.021, GFI = 0.933, AGFI = 0.901, NFI = 0.905, IFI = 0.941, TLI = 0.925, CFI = 0.940, RMSEA = 0.067. Furthermore, the standardized factor loading was over 0.5, and the conceptual reliability was over 0.7, indicating that the results were statistically significant [72].

#### 4.2.2. Discriminant Validity

Convergent validity and discriminant validity were proven successfully. It can be said that there is discriminant validity when the average variances extracted (AVEs) for the two constructs are greater than the square of the correlation coefficient for said constructs. Discriminant validity is demonstrated by the data presented in Table 3 [73]. A formularization of the relationship between “workaholism” and “work–family conflict”, which has the highest correlation coefficient of any of the variables, demonstrates the correlation coefficient for workaholism and work–family conflict to be 0.554, which means (0.554)^2^ = 0.306. The AVEs for workaholism and work–family conflict are 0.617 and 0.607, respectively. Not only were the AVEs for both variables greater than the square of the correlation coefficient, but the AVE for job insecurity was also greater than 0.306. Consequently, the results were shown to demonstrate discriminant validity.

### 4.3. Hypothesis Testing

**Figure 2:**
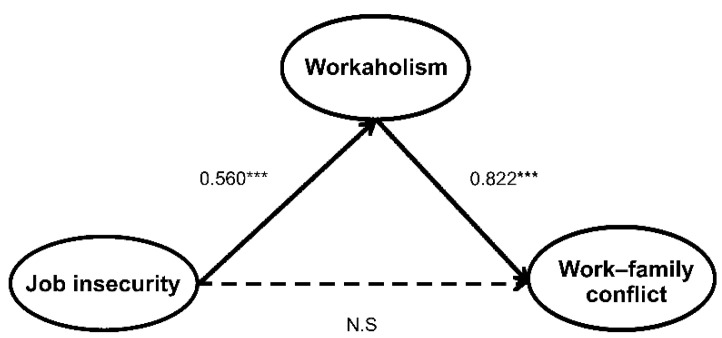
Structural equation model with parameter estimates. *** *p* < 0.001. Nonsignificant paths are shown in dotted lines.

This study implemented structural equation modeling using AMOS 18.0 to test the hypotheses. Given the results of χ^2^ = 154.187(df = 62, *p* = 0.000), CMIN/DF = 2.487, RMR = 0.021, GFI = 0.933, AGFI = 0.901, NFI = 0.905, IFI = 0.941, TLI = 0.925, CFI = 0.940, RMSEA = 0.067, it was concluded that the model fit is acceptable [72]. The data in Table 4 and Figure 2 represent the results of the hypothesis testing. Further, job insecurity was found to have a positive effect on workaholism (β = 0.560, *p* < 0.001), thus supporting Hypothesis 1. Workaholism was found to have a positive effect on work–family conflict (β = 0.882, *p* < 0.001), thus supporting Hypothesis 2. However, job insecurity was not found to have positive effect on work–family conflict (β = 0.013, *p* > 0.05), thus not supporting Hypothesis 3. As an indirect effect of adopting 500 bootstrap samples to test Hypothesis 4, job insecurity was shown to have a positive correlation with work–family conflict through the mediation of workaholism (β = 0.460, *p* < 0.01).

## 5. Discussion

This study recognizes that hotels are fundamentally reshaping the labor market by downsizing or attempting mergers to survive amid rapid social and economic changes. It was conducted for the purpose of determining the correlations between workaholism, work–family conflict, and the job insecurity perceived by hotel workers, subsequently providing reference material for managing hotel workers.

The results of the empirical analysis of this study are as follows. The testing of Hypothesis 1 confirms that the job insecurity perceived by hotel workers has a direct effect on workaholism and supports the conclusions of previous studies that workaholism has an effect on its sub-factors—work commitment and the compulsive drive to work [28,29]. The reason is thought to be that the greater hotel workers’ perception of job insecurity, the more that they increase their workload because they wish to be considered as valuable assets by their business managers and avoid finding themselves in a risky employment situation. As such work commitment is not voluntary but a product of job insecurity, it is expected to have a negative impact on the services and productivity of hotels in the long run. Given that job insecurity also has a direct impact on the compulsive drive to work, hotel business managers should devise a plan to mitigate their workers’ job insecurity by building employee capacity and thereby enhance the competitiveness of their hotels.

The testing of Hypothesis 2 demonstrates that the job insecurity perceived by hotel workers has a direct effect on work–family conflict, which reinforces the findings of previous studies [41,42,74]. Concerns such as anxiety from increased workload and the continuity of work can be said to trigger various types of work–family conflicts. To prevent this, hotel business managers should set clear working hours and devise a solution that improves the conditions of working hours.

The testing of Hypothesis 3 shows that the perceived job insecurity of hotel workers does not affect work–family conflict, conflicting with the findings of previous studies [55,69,70]. Nevertheless, the correlation analysis revealed that there is a positive correlation between the two variables, indicating the presence of a meaningful influence.

The testing of Hypothesis 4 confirms that job insecurity affects work–family conflict through the mediation of workaholism. Under unfavorable conditions, workers affected by job insecurity are thought to experience increased work commitment because of a compulsive drive to work. This aspect, in turn, is believed to ultimately have a negative effect on work–family conflict.

To sum up the results of the hypotheses, the job insecurity perceived by the hotel worker increases workaholism and causes conflicts between work and family.

This study is expected to have the following implications. First, as one of the early studies on hotel workers in the country, where there is relatively little research on workaholism, it is expected to proliferate more related studies on the subject. In particular, it is expected to contribute to the development of academic theories by verifying the causal relationships between job insecurity, workaholism, and work–family conflict observed among hotel workers.

Second, the important implications of this study, the subjects of which were hotel workers who consider top-notch human services to be the most important product component, are expected to pave the way for new ways of managing the mentality and working hours of workers. In particular, they are expected to provide an opportunity to explore measures for preventing several negative causes of job insecurity.

Third, this study offers a solution for building a system that enhances the overall quality of life of workers by encouraging the social responsibility of hotel business owners and presenting a practical solution.

In light of the limitations, which are mentioned below, this study proposes that follow-up studies be conducted in the future. The first limitation is related to the selection of survey participants. This study surveyed only those hotel workers who worked in four-star hotels or above in metropolitan areas. Moreover, the survey was conducted without distinguishing permanent employees from temporary workers. A greater regional distribution and the differentiation of employment statuses are expected to help derive more detailed findings in the future. The second limitation is that every worker may perceive job insecurity differently depending on his or her own circumstances, as perceived job insecurity hinges on the subjectivity of each worker. Consequently, conducting a study that compensates for the above limitations of this study is expected to help draw more useful conclusions. It is thought that policy and legislation on improving the working environment of workers are needed.

## 6. Conclusions

Based on our results, it can be concluded that with the changes to the landscape of the labor market, the idea of lifetime employment has become outdated. Workers now live in a new era where they may be laid off at any moment. This is true not just for hotel workers but also for workers in other industries, and companies should develop a solution for alleviating their employees’ anxiety about job insecurity. As evidenced by the findings of this study, job insecurity is deemed to have a negative impact throughout workers’ lives because it has a direct effect on workaholism, which ultimately results in an unstable work and home lives. As such, hotels should improve working conditions and propose solutions, such as the moderation of their workloads, for preventing their workers from becoming workaholics. In particular, hotel business managers should minimize workers’ job-insecurity-induced compulsive drive to work by devising strategies for minimizing their workers’ workloads. They should also enable workers to perform their jobs autonomously. As family conflict has a direct relationship with life satisfaction, hotel managers should devise a solution that allows rewards and incentives to directly benefit their workers’ family members as well. This study presents theoretical and practical implications for human resource management by clarifying the relationship between job insecurity, workaholism and work–family conflict of hotel employees who are vulnerable to changes in external and internal environment.

## Figures and Tables

**Table 1:** Demographic factors of the participants.

Demographic Factors	Category	Number of Participants	Percentage (%)
Gender	Male	217	65.6
	Female	114	34.4
Age	20s	143	43.2
	30s	125	37.8
	40s	55	16.6
	50s and older	8	2.4
Education	High school diploma or less	23	6.9
	Associate degree	193	58.3
	Bachelor’s degree (4-year university)	91	27.5
	Graduate degree or higher	24	7.3
Experience	Less than 5 years	150	45.3
	Between 6 and 10 years	79	23.9
	Between 11 and 16 years	50	15.1
	Between 15 and 20 years	41	12.4
	20 years or more	11	3.3
Department	Rooms division	75	22.7
	Sales and marketing	40	12.1
	Finance	31	9.4
	Engineering	21	6.3
	Executive office	53	16.0
	Food and beverage	88	26.6
	Human resources	19	5.7
	Security	4	1.2
Total		331	100

**Table 2:** Confirmatory factor analysis.

Factor and Variable		Standardized Loading	S.E	C.R	AVE	Composite Construct Reliability(CCR)	Cronbach’s α
Job insecurity	JI1	0.704	-	-	0.666	0.909	0.847
	JI2	0.724	0.099	11.661			
	JI3	0.732	0.094	11.776			
	JI4	0.712	0.102	11.495			
	JI5	0.756	0.097	12.097			
Workaholism	Work commitment	0.582	-	-	0.617	0.763	0.826
	Pressure	0.616	0.123	7.616			
Work–family conflict	W-FC1	0.682	-	-	0.607	0.902	0.832
	W-FC2	0.719	0.098	11.231			
	W-FC3	0.608	0.092	9.712			
	W-FC4	0.672	0.086	10.602			
	W-FC5	0.702	0.092	11.000			
	W-FC6	0.666	0.101	10.514			

χ^2^ = 154.187 (df = 62, *p* = 0.000), CMIN/DF = 2.487, RMR = 0.021, GFI = 0.933, AGFI = 0.901, NFI = 0.905, IFI = 0.941, TLI = 0.925, CFI = 0.940, RMSEA = 0.067.

**Table 3:** Discriminant validity of the variables.

Factor	Job Insecurity	Workaholism	Work-Family Conflict
Job insecurity	**0.666** ^1^	0.144 ^3^	0.161
Workaholism	0.380 **^,2^	**0.617**	0.306
Work–family conflict	0.402 **	0.554 **	**0.607**
Mean	3.80	3.80	3.88
S.D.	0.589	0.485	0.544

** *p* < 0.01; ^1^ Diagonal: Average Variance Extracted (AVE); ^2^ Area below diagonal: The correlation coefficient for the constructs (r); ^3^ Area above diagonal: The square of the correlation coefficient (r^2^).

**Table 4:** Results of structural equation model analysis.

Process (Hypothesis)		Beta	*t*-Value	*p*-Value	Indirect Effect		Decision
					Coefficient	*p*	
H1	Job insecurity → Workaholism	0.560	6.087 ***	0.000			Accepted
H2	Workaholism → Work–family conflict	0.822	4.970 ***	0.000			Accepted
H3	Job insecurity → Work–family conflict	0.013	0.119	0.905			Rejected
H4	Job insecurity → Work–family conflict (the mediating effect of workaholism)	0.013	0.119	0.905	0.460 **	0.004	Accepted

** *p* < 0.01; *** *p* < 0.001; χ^2^ = 154.187 (df = 62, *p* = 0.000), CMIN/DF = 2.487, RMR = 0.021, GFI = 0.933, AGFI = 0.901, NFI = 0.905, IFI = 0.941, TLI = 0.925, CFI = 0.940, RMSEA = 0.067.
